# Motivational Interviewing (MI) in Obesity Care: Cultivating Person‐Centered and Supportive Clinical Conversations to Reduce Stigma: A Narrative Review

**DOI:** 10.1002/osp4.70057

**Published:** 2025-02-10

**Authors:** Violeta Moizé, Yitka Graham, Ximena Ramos Salas, Mercè Balcells

**Affiliations:** ^1^ Obesity Unit Endocrinology and Nutrition Department Hospital Clinic Barcelona Barcelona Spain; ^2^ Department of Medicine University of Barcelona Barcelona Spain; ^3^ Translational Research in Diabetes, Lipids and Obesity Consortium August Pi i Sunyer Biomedical Research Institute (IDIBAPS) Barcelona Spain; ^4^ Grupo en Español de Trabajo de Entrevista Motivacional (GETEM) Barcelona Spain; ^5^ Faculty of Health Sciences and Wellbeing Helen McArdle Nursing and Care Research Institute University of Sunderland Sunderland UK; ^6^ Bariatric Surgical Unit Sunderland Royal Hospital South Tyneside and Sunderland NHS Foundation Trust Sunderland UK; ^7^ Faculty of Psychology University of Anahuac Mexico City Mexico; ^8^ Faculty of Biomedical Science Austral University Buenos Aires Argentina; ^9^ Bias 180 Ontario Canada; ^10^ Replica Communications Kristianstad Sweden; ^11^ Addictive Behavior Unit Psychiatry and Psychology Service Clinical Institute of Neurosciences (ICN) Hospital Clínic Barcelona Barcelona Spain; ^12^ Addiction Research Group Consortium August Pi i Sunyer Biomedical Research Institut (IDIBAPS) Barcelona Spain

**Keywords:** collaborative approach, continued support, heath care professional experience, patient experience, patient values, positive communication

## Abstract

**Background:**

Patients perceive high levels of weight prejudice, stigma, and discrimination within health systems, affecting their ability to manage their obesity and related chronic conditions. Scientific and patient obesity associations worldwide have prioritized the reduction of weight stigma to improve patient experiences in health systems and overall health outcomes. Since a significant proportion of the population is now living with multiple chronic diseases related to obesity, healthcare systems must shift toward multi‐disease management frameworks incorporating person‐centered and non‐stigmatizing clinical conversations. Motivational Interviewing (MI) has the potential to transform clinical interactions by using non‐stigmatizing language, communication, and practices. Studies using MI in obesity management have solely focused on weight loss outcomes, while other patient experience related outcomes would also be relevant to evaluate.

**Methods:**

A narrative review was undertaken to critically analyze the potential impact of MI on obesity and chronic disease management practices and experiences.

**Findings:**

An analysis and contextualization of the MI theoretical framework for obesity management, based on the philosophy of motivational spirit, was reviewed, assessing micro skills or strategies.

**Conclusion:**

MI may assist healthcare professionals conduct non‐stigmatizing clinical conversations in accordance with basic principles of collaborative therapeutic alliances. A proposal for research considerations that can help illuminate the potential for of MI in obesity management is also outlined.

## 1 Introduction

### 1.1 Overview of Stigma and Patient Centered Approach

Amongst psychosocial issues relating to health, stigma is an independent driver of inequalities, which needs to be prioritized and addressed [1]. Conceptualized by Goffman in 1963, stigma is defined as a condition, attribute, trait, or behavior that causes a person to be included in a social category that is seen as unacceptable or inferior [2]. The consequences of these negative attributes, which broadly encompass characteristics such as race, ethnicity, religion, physical deformities and perceived character flaws, result in a devaluation of an individual or group [3]. Through social norms and practices, stigma is reinforced within communities, influencing how we see stigmatized groups and ourselves as well as by creating language and antagonistic relationships (e.g. sick/healthy; doctors/patients; adolescents/adults) [3]. Stigma exists in a wide range of social settings including education, employment and worryingly, in healthcare settings [4, 5].

Broadly speaking, there are several types of stigma which have been posited: external/public stigma, internal/self‐stigma, and institutional stigma (see Table 1).

**TABLE 1:** Types of stigma.

	External/public	Internal/self	Institutional
Stereotype	Negative beliefs about a group (e.g., dangerousness, incompetence, character weakness)	Negative beliefs about the self (e.g., character weakness, incompetence)	Stereotypes are embodied in laws, policies, and other institutional practices
Prejudice	Agreement with stereotypes, negative emotional reaction toward stigmatized individuals or groups (e.g., feels anger or fear toward the group)	Agreement with stereotypes, negative emotional reaction toward oneself (e.g., low self‐esteem, low self‐efficacy—e.g. “I must be lazy if society thinks I am”)	Agreement with stereotypes, negative emotional reaction toward stigmatized individuals or groups (laws or policy that disadvantage stigmatized individuals or groups)
Discrimination	Behavior response owing to prejudice (e.g., avoidance, withhold opportunities such employment or fail to offer help)	Behavior response to prejudice (e.g., social isolation; delay or avoidance of healthcare services)	Intended or unintended loss of opportunity (e.g. discriminatory hiring policies in workplaces or discriminatory healthcare practices such as denial of disease screening, diagnosis, or treatment)

### 1.2 Health‐Related Stigma

Health‐related stigma or discrediting attitudes toward diseases and conditions such as mental illness, HIV/AIDS, substance use, and obesity are considered independent social determinants of health [6]. Health related stigma can also intersect with other types of stigmas such as racialized identities, sexual orientation, gender identity, and age [7, 8].

“Stigma’s impact on a person’s life may be as harmful as the direct effects of the disease” [9].

Stigma can elicit emotions such as fear, disgust, anger, pity, or empathy, with emotional responses to stigmatized attributes influencing behavior such as avoidance of stigmatized individuals and groups [3]. People living with stigmatized conditions may avoid help‐seeking and clinical encounters, have lower adherence with treatments, and experience suboptimal physical and mental wellbeing in addition to other factors which may affect health and socioeconomic outcomes [10]. Health‐related stigma drivers such as misinformation and lack of knowledge about the causes of diseases and conditions and stereotypes about individuals living with these conditions can create unequal power dynamics, which may lead to stigmatizing healthcare practices and policies [8].

Healthcare professionals’ discrediting attitudes toward stigmatized diseases and conditions can convey a sense of devalued status to patients and create a lack of trust. This lack of trust can negatively impact clinical conversations when, for example, patients do not share all the necessary information required to agree on a disease management plan, which ultimately impacts overall health outcomes.

The conveying of a devalued status to patients by healthcare professionals is increasingly recognized as a crucial factor in perpetuating health related stigma, particularly with regard to mental health [11] and obesity [12]. Obesity stigma is not challenged as often as other forms of health‐related stigma and is often portrayed as a beneficial incentive for behavior change and weight loss [13], reinforcing internalized stigma and further reducing the quality of life for people living with obesity.

### 1.3 Obesity Stigma

Negative beliefs and attitudes about individuals because of their weight (i.e. weight bias) and social stereotypes or prejudice toward individuals with a higher weight (i.e. weight stigma) can interact and influence obesity management clinical communications and patient‐provider interactions [12]. For instance, healthcare professionals who tacitly view a patient's obesity as their own fault can lead to inadequate obesity management support and care or even rejection of evidence‐based obesity treatment. Weight stigma experiences in healthcare settings can also impede patient care‐seeking and intensify unfavorable health behaviors (e.g. adoption of unevidenced‐based weight loss practices that can worsen obesity and related complications) [14, 15].

Research indicates that weight stigma can also be a barrier to accessing treatments for obesity and obesity related chronic diseases such as diabetes, hypertension, anxiety, and depression [14, 15]. Numerous studies demonstrate that patients living with obesity report being treated with less respect and feel judged for their weight by healthcare professionals. For example, in a recent Canadian study, 38.1% of patients living with obesity and at least one other chronic disease perceived that healthcare professionals make assumptions about their eating and physical activity because of their weight [14].

Some of patients’ perceived weight stigma experiences from healthcare professionals seem to be associated with the language that healthcare professionals use during clinical interactions [16]. By identifying and addressing the stigmatizing processes involved in these clinical interactions, it may be possible to improve access to and the quality of obesity care services and ultimately improve patient experiences and obesity treatment outcomes.

This perspective allows us to broaden the focus of obesity stigma interventions, which, to date, have mostly been focused on modifying beliefs about obesity through education [17, 18] as well as promoting the acceptance and recognition of obesity as a chronic disease [17, 19]. Scientific and patient obesity associations worldwide have prioritized the reduction of weight stigma to improve health care services for people living with obesity [20, 21]. Some of the strategies used by the obesity community to reduce weight bias, stigma and weight‐based discrimination, include: educating healthcare professionals [22, 23, 24, 25], changing the definition of obesity to distinguish between body size and adiposity related health impairments and shifting the focus of obesity treatment outcomes to health and wellbeing rather than just weight loss [26, 27], raising awareness about the importance of using non‐stigmatizing language in obesity research healthcare and public policy [28, 29, 30, 31], advancing legislative policies against weight‐based discrimination [32], changing the portrayal of individuals living with obesity in the media [33], and promoting a paradigm shift in health promotion programs away from weight‐centric strategies [34].

Motivational interviewing (MI) has the potential to transform clinical interactions by using non‐stigmatizing language, communication, and practices. This approach emphasizes patient empowerment, fostering more open and positive clinical encounters for individuals living with obesity.

Patient empowerment and stigma are two contrasting concepts in healthcare that significantly impact patient experiences and outcomes. Patient empowerment has gained prominence in healthcare, as part of a move away from paternalism toward more equitable and collaborative models of healthcare delivery [35]. This has the potential for improving the cost‐effectiveness of care, especially for people affected by chronic conditions [36].

There is an increasing shift in clinical in weight management to put the patient at the center of care, and empower them to become active, rather than passive recipients of their care, with shared decision‐making between patient and professional the aim [37]. Non‐compliance with interventions, may be the result of a patient's lack of psychological skills to engage with and develop healthy weight behaviors [38]. Although behavioral and psychological interventions for weight management are not new, there is increasing evidence which positions behavior‐based interventions such as acceptance and commitment therapy (ACT) and MI, which focus on commitment [39], may have an important role to play in patient empowerment in the context of obesity treatment. Our review focuses on the potential of motivational interviewing (MI) as an opportunity to develop meaningful conversations between patients and professionals, where the impact of stigma can be explored and strategies can be developed to reduce its impact on positive behavior changes related to weight management, broadening the focus to include aspects beyond weight loss.

### 1.4 Overview of Motivational Interviewing

MI is a style of direct, patient‐centered assistance that brings about behavior change by helping to explore and resolve ambivalence [40]. MI is grounded in the premise that people are not unmotivated to change their behaviors but rather, they are ambivalent to change because the behaviors do not align with their values or goals [41]. In the context of chronic disease management strategies that may require behavior change, it is important for healthcare professionals to recognize this ambivalence and support patients to address their individual behavior change barriers so that clinical treatment strategies are not seen as a challenge to a person's freedom, while also empowering patients to make behavior changes that align with their values or goals [42].

MI was born at a time when evidence‐based medicine was particularly relevant and articles exploring and validating its efficacy rapidly appeared in multiple publications. The founders of MI, Miller, and Rollnick, posited that the method offers a therapeutic style which is evidence‐based, to co‐deliver other treatments more effectively, and based on more than 40 years of practice [41, 42, 43].

Miller systematized the learnings from his work in the field of addictive behaviors in his first book on Motivational Interviewing, presenting a straightforward and pragmatic, yet novel approach to the treatment of addictions, which had previously been treated with confrontational and hierarchical models [40]. There is now significant scientific evidence demonstrating the effectiveness of MI in improving the treatment of multiple addictive disorders, especially alcohol use disorders [43, 44, 45], smoking cessation [46, 47] and cannabis cessation [48], as well as for pathological gambling [49, 50, 51, 52].

The first MI meta‐analyses [53, 54, 55, 56] (despite the heterogeneity of the articles included) reached three main conclusions: (a) MI is an approach that increases treatment retention, making it easier for the patient to return to the next visit and increase the probability for behavior change; (b) MI increases adherence to treatment, favoring adherence with the agreed indications prescribed during the interview; and (c) MI increases the engagement of the healthcare provider (doctor, therapist, nurse, etc.) in the treatment plan.

In this review, the principles of MI will be discussed, that is, collaboration, acceptance, empowerment, and compassion, which may be crucial to be able to establish a non‐stigmatizing therapeutic relationship that ensures good care for people living with obesity. Motivational interviewing techniques may not only optimize therapeutic interactions and facilitate person‐centered obesity care but could also help address internalized weight stigma that many patients living with obesity may have.

As the interest in Motivational Interviewing has grown, its areas of application are expanding in other areas such as: health behavior change, cardiovascular diseases, endocrine disorders such as diabetes, eating disorders, HIV infection prevention, therapeutic adherence, health promotion strategies such as nutrition and physical activity interventions, pathological gambling, affective disorders, first psychotic episodes, and the long‐term management of chronic diseases among others. A 2013 systematic review and meta‐analysis of randomized controlled trials evaluating the efficacy of Motivational Interviewing in healthcare settings concluded that, overall, patients who receive Motivational Interviewing‐based interventions are 1.5 times more likely to improve on a wide range of health outcomes (such as physical activity, HIV viral load, blood pressure, and serum cholesterol) compared to control groups [45].

### 1.5 Motivational Interviewing in Obesity Management

In the treatment of obesity, MI has also been investigated in adults [53], children [54] and families [55]. However, none of these studies assessed the impact of MI on outcomes beyond weight loss. The effectiveness of MI in terms of improving patient experiences in obesity management, treatment adherence, quality of life, or reducing weight stigma has not yet been studied [56].

### 1.6 The Argument for MI to Improve Patient Experiences of Obesity Care

Obesity is a complex chronic disease caused by many intersecting factors including biological (e.g., genetics, neurohormonal factors), psychological (e.g. depression, mood disorders), medical (e.g. weight promoting medications), socioeconomic (e.g., education, income, employment, social deprivation, inequalities, economic policies), and environmental (e.g., unhealthy food and physical activity environments) factors [57]. Unfortunately, obesity is often simplified as a lifestyle risk factor or a behavioral problem. This simplification of obesity ignores the biological aspects of the disease, which are not within an individual's control and places the entire responsibility for obesity prevention and management on the individual [58]. There is scientific and clinical consensus that obesity should be managed using basic principles of chronic disease care using non‐stigmatizing and evidence‐based treatments [59]. Since obesity is a heterogenous disease (i.e. people develop obesity for many different reasons), obesity requires an individualized treatment plan that addresses the specific barriers for each patient, while reflecting the person's specific realities, resources, and preferences [27]. Evidence‐based treatments for obesity include behavioral and psychological interventions, pharmacotherapy, and bariatric surgery [27]. These three evidence‐based treatments can support healthy nutrition and physical activity, which are foundational strategies for the prevention and management of chronic diseases, including obesity.

As with any other chronic disease management plan, patients need to engage in various behaviors such as healthy eating, physical activity, medication adherence, and attending regular medical appointments, etc. Therefore, it is essential for obesity management plans to include support for behavior change. This requires a new patient‐healthcare professional therapeutic model that is collaborative and attuned to patients' psychosocial realities, rather than a confrontational and hierarchical model that has been used traditionally [60].

There are three ways in which MI may contribute to improving obesity management experiences for patients living with obesity:

- MI is a collaborative interviewing style, aimed at enhancing the other person's capacities and his/her own motives for change; it can help healthcare providers understand patients' capacities and realities to engage in obesity management interventions.
- MI is a person‐centered therapeutic approach allowing healthcare professionals and patients to explore and resolve the barriers that accompany any change process. Chronic disease management requires change processes that need to be explored and resolved in collaboration with patients and healthcare professionals.
- Since MI is a collaborative, goal‐directed communication style that puts a selective focus on the language of change, it can strengthen the motivation to change by both healthcare professionals and patients by exploring and evoking their individual motives to change.

### 1.7 Operationalizing MI in Obesity Care

The MI theoretical framework is grounded within the Motivational Spirit philosophy and is operationalized through a series of micro skills or strategies that can help healthcare professionals to conduct a clinical conversation in accordance with the basic principles of collaborative therapeutic alliances [40, 61].

The Motivational Spirit has 4 components: (1.) *Partnership (*vs. *paternalism):* Establishing a collaborative relationship between the healthcare provider and the patient. This involves working together as equals and building a sense of trust and mutual respect. (2.) *Acceptance (*vs. *imposition):* Demonstrating a non‐judgmental attitude toward the patient. This component encompasses four aspects: absolute worth (respecting the patient's inherent value), accurate empathy (understanding the patient’s perspective), autonomy support (respecting the patient's right to make their own choices), and affirmation (acknowledging the patient's strengths and efforts). (3.) *Compassion (*vs. *indifference):* Prioritizing the patients well‐being and acting in their best interest. This means actively listening, showing genuine concern, and supporting the patient's goals and values. (4.) *Empowerment (*vs. *education):* Drawing out the patient’s own motivations and resources for change. This involves eliciting the patient's ideas, reasons, and desires for making positive changes, rather than imposing the provider's views or solutions [40]. The specific MI skills or strategies are described in Table 2:

**TABLE 2:** Motivational interview skills.

Skill	Theoretical framework	Example
Open questions	An open‐ended question is one that cannot be answered with a “yes” or “no” or in a few words. An open question invites reflection before answering and offers a range of possible answers, while a closed question limits the range of answers and leads to a short answer.	*“What brings you here today?” or “would you like to tell me more about your experience with living with obesity?”*
		*Can you tell me more how you felt in your patient journey in relation to the stigma associated with obesity?*
Affirmations	To allow healthcare professionals to recognize and support patients' strengths and efforts while helping them to work on their self‐efficacy toward change.	*“You've really put a lot of effort into changing your sleep habits,” or “It sounds like you've been very dedicated to your stress management routine.”*
		*I understand how frustrating it must be when you do not feel trusted, and feel blamed for your weight gain… Many people would have stopped going to the doctor in your situation and I thank you for the trust.*
Reflective listening	This involves actively listening to the patient and then reflecting back what you've heard. It helps to ensure understanding and shows empathy. There are different levels of reflection, from simple reflections (repeating or rephrasing what the patient said) to complex reflections (adding meaning or emphasizing emotional content).	*“You're feeling frustrated because it's hard to find time to attend your medical appointments/support group sessions with your busy schedule.”*
		*You're afraid that professionals will blame you for the weight regain you're experiencing.*
Summarizing	The summary gathers information that the patient gives us and allows us to narrow down the discourse of very scattered patients, as well as to check that we are understanding each other	*“So, let me summarize what we've discussed today. You're feeling good about your progress with your stress management plan but finding time to attend medical appointments and/or support group sessions is still a challenge and you want to find a schedule that fits you.”*

MI is a relationship‐building encounter and a communicative style that can be used in a therapeutic/clinical conversation or interaction, and not a therapeutic intervention. The idea is that a clinical interaction could be improved if the spirit and style of the communications is person‐centered, empathetic, non‐stigmatizing, focused, and evoking of patients' own intrinsic motivations and capabilities to meaningfully implement the changes required as part of their obesity management plan.

The process by which MI could theoretically improve patients’ experiences with obesity management can be summarized into four phases: (a) Engaging, (b) Focusing, (c) Evoking, and d) Planning.

#### 1.7.1 Engaging

Engaging is the process of establishing a helping relationship based on mutual respect and trust (therapeutic alliance). Starting a clinical conversation or interaction without engaging with the patient hinders patients’ sense of security and trust, which would negatively impact the ability of healthcare providers to explore and resolve patients’ barriers to obesity care plans.

Healthcare professionals can assess whether they are creating the necessary bond with patients by reflecting on the following questions:

**Table:** 

Does my patient feel comfortable talking to me?
Have I generated an empathetic and supportive clinical environment?
Is this clinical interaction collaborative between myself and the patient?
Do I understand my patient's point of view and concerns?

Patients can assess whether their interactions with their healthcare providers are respectful and collaborative by reflecting on the following questions:

**Table:** 

Does the healthcare professional listen to me and understand me?
Do I feel like I can trust this healthcare professional?
Can I safely and openly express my opinion on what happens in the consultation? Does he/she offer me options or let me choose?
Does he/she negotiate, or can I agree on what to do?

Active Listening is a key strategy used in MI to be able to bond with patients. Active listening requires a healthcare provider to check that they have understood what patients are sharing with them [62, 63]. Key MI techniques for active listening include: (a) Rephrasing where healthcare professionals can repeat an piece of information that a patient has said using synonyms or altering it slightly just to clarify, and (b) paraphrasing where healthcare professionals infer what the patient has said using new words, broadening the perspective of what has been said [63, 64].

#### 1.7.2 Focusing

Focusing is the process of seeking, finding, and maintaining the direction of the clinical conversation or interaction.

Healthcare professionals and patients often have a specific goal for a clinical conversation or interaction. These goals may be different. The process of focusing aims to clarify clinical conversation goals [64].

During the focusing process, healthcare professionals should ask themselves if they are correctly identifying the patient's medical concerns and goals. Understanding the patient's goals is necessary to have a clear idea of where the clinical interaction is heading [65]. By focusing, the clinical encounter can feel more like “dancing” as opposed “boxing/fighting” with the patient (Are we dancing or boxing?) Focusing is a key process in the emergence of clinical discordance. The intensity of clinical discordance is directly proportional to the distance between the patient's goals and those proposed by the healthcare professional [38].

In trying to understand clinical discordance, it is important for healthcare professionals to consider patients’ feedback during a clinical conversation. A discordance between patient‐provider goals and indicates a need to change the approach, shifting toward understanding patients’ goals more clearly and working toward shared therapeutic goals without confrontation. Sometimes patients’ goals are not clear and the focusing process can help healthcare providers explore a person’s goals and values and to identify and agree on the direction to follow [66]. Values are part of a person's beliefs, and they express a person’s interests or feelings as well as determine their behavior [67]. A discrepancy can exist between a person’s current behavior and their personal values. Discrepancies are intrinsic to the patient and have to do with their internal scale of values and healthcare professionals cannot impose them. Healthcare professionals can only facilitate that the discrepancies become visible and appear in the therapeutic process as they are an important driver of change [40, 61].

It may happen that the person's values do not coincide with a healthcare professional's values. This does not need to interfere in the therapeutic relationship since respect and acceptance are key factors that support patients in their process of change and disease management. Healthcare professionals can discuss with their patients that knowing and accepting our own values can be helpful in any chronic disease management process [65, 67].

Ultimately, everyone has their own reasons for change when it comes to chronic disease management and the role of healthcare professionals is to assess patients' internal frame of reference by understanding their goals and values (Table 3).

**TABLE 3:** Example of questions that help patients understand their own values and preferences.

Focusing	What really matters to you?
Evoking	What are your real reasons for change?
Planning	What are the strategies that can best fit your values?

#### 1.7.3 Evoking

As Blaise Pascal quoted, ‘Generally people are more convinced by the reasons they discover for themselves, rather than by those explained to them by others.’

To evoke is to extract from the patient his own motives and capacities to change. It is the most important process of MI and occupies a large part of the clinical conversation or interaction [40].

MI places a selective focus on the language of change. MI is specifically aimed at strengthening the motivation to change by exploring and evoking a person's own individual reasons for change. It is in this process of evocation that we must explore not only the individual's own motives for change but also his or her capacities and abilities to change [40]. MI moves away from the “deficit model” where the role of healthcare professionals is to educate patients about what they need to do. Instead, the MI approach is to activate or highlight a person’s strengths and capacities [40]. Healthcare professionals who have already created a bond and have correctly focused on the treatment goals, will find in the evocation process the opportunity to collaborate with their patients based on their own capacities and experiences, thus facilitating changes necessary for obesity care plans [41]. In short, the MI approach focuses on creating greater patient empowerment, which is a key factor in chronic disease management.

Listening to patients’ ideas and suggestions is essential to adapt interventions for obesity which can be aligned to their lifestyle, beliefs, and personal context to improve treatment outcomes. MI differs from other psychotherapeutic approaches in that it is more directive, although not in the sense of telling the person what he/she should do, but in directing clinical conversations and interactions to empower patients to decide how, when and in what way they want to make changes as part of their obesity treatment plan. Healthcare professionals should be attentive to signs in terms of words and phrases that patients use which indicate they are preparing for change. The discourse of change is, in its initial phases, one of preparation for change (preparation, desire, skills, reasons, and need for change) to progressively reach a discourse of mobilizing change (commitment, activation, and initiation of change).

Healthcare professionals should additionally facilitate and direct clinical conversations and interactions toward this discourse of change that should come from the patient. A listening attitude that allows the healthcare professional to see and understand a patient's reality will be extremely useful to strengthen and reinforce behavior change.

#### 1.7.4 Planning

Planning is the last of the four phases in the MI process and refers to the part of the clinical conversation and interaction in which, through active patient participation, goals are established, options are evaluated, and a plan of action is developed [40].

The four phases are somewhat sequential or linear. Creating a bond with patients goes first and identifying clear goals is a prerequisite for evoking and planning. But at the same time, the four phases are also recurrent. A bond is established from the beginning, but the relationship with a patient must be nurtured throughout the MI process and evocation can also be a part of the clinical conversation or interaction from the very beginning. Likewise, agreeing on a clear goal is not a static process; it may require several focusing steps and the goal may change and adapt throughout the obesity therapeutic journey.

But for the purpose of simplifying the four MI phases, the process may be operationalized as follows:

1. The first step is for healthcare professionals to create a bond with the patient using the MI spirit and style of communication, which includes person‐centered and empathic listening (*Linking and Evoking*).
2. The second step involves identifying a clear therapeutic treatment goal by focusing the clinical conversation on patients' goals and values. (*Focus*).
3. The third step is for healthcare professionals to evoke a patient's intrinsic motivation (or plans) to change as part of their obesity treatment plan (*Evoking*)
4. The fourth step occurs when the patient decides to undergo behavior change and makes the change into action, deciding goals and strategies to achieve these (*Planning*).

The four processes of MI are engaging, focusing, and evoking; although the planning process is not always reached, in MI, the Planning process is optional. The primary goals in MI are to create a good working relationship with the client, identify specific target behaviors, help the client/offender to build motivation toward these target behaviors by using specific skills and strategies, and work toward aiding the client/offender in resolving their ambivalence and choosing change. Planning encompasses both developing a commitment to change and developing a specific plan of action (goal setting; sorting options; forming plans; building support) [40].

When to move from evoking to planning depends on healthcare professionals' own clinical judgment guided by a patient’s signals of readiness. Signals that a patient is ready to move into the planning phase can include increased change talk and decreased status quo talk, resolution of barriers, visualization of change, and initiation of first steps toward the new behavior related to their obesity care plan [68].

Once the signs have been detected and have previously evoked the patient’s values, capacities and skills, a change plan can be created, enhancing those strategies that fit better and in a realistic way with the patient's life. In the creation of a change plan, healthcare professionals can exchange information with the patient in a bidirectional way and investigate what the patient is interested in and what he/she knows in order to adjust the information he/she will need to jointly create a change plan [40].

### 1.8 Information Exchange as Key Strategy in the Planning Process

With permission to talk about weight, a non‐judgmental (another core principle of motivational interviewing) conversation is more likely. Non‐judgmental curiosity helps avoid challenges to effective communication. It is important not to make assumptions about the patient's lifestyle; many people living with obesity might already be working hard at weight management. The 5 As (ask, assess, advise, agree, and assist), developed for smoking cessation, can be adapted for obesity counseling [69]. Prior studies focused on training professionals on MI to address obesity to improve the patient‐centered approach [70, 71, 72, 73, 74, 75]. Although they show that MI could be effectively incorporated in clinical practice, weather these approaches improve patient experience and health related outcomes beyond weight haven't been yet addressed.

Many healthcare professionals tend to overestimate the amount of information they must transmit to a patient, erroneously believing that this will help a patient to make decisions regarding the change [40, 68]. It should be reinforced that patients themselves are a valuable source of information that facilitates the creation and adjustment of a change plan that matches their capabilities and daily life in a realistic way [76].

One strategy that can facilitate bi‐directional information exchange is called Question‐Information‐Question or QIQ. The QIQ strategy consists of always giving the information preceded by an open question and asking permission. Examples of this include: What would you like to know? May I give you information about some aspects of…? Would you be interested in talking about…? What do you know about…? What have you been told about…?.

If a patient gives permission to provide them with more information or shows interest in receiving more information about weight management, healthcare providers can provide the information in a manner that is accepting and understood by the patient, which also allows time for reflection by the individual. Healthcare professionals must also present the information ensuring that any information does not contain stigmatizing language, and that they cognizant of the patients’ right to ignore or disagree with the information [65]. It is also helpful to end the consultation with a question to see if the information given has been understood and if the patient is satisfied with the encounter.

The last phase in the MI process is active patient participation, in which goals are established, options are evaluated, and an action plan is created, establishing priorities in the changes to be achieved, with objectives in accordance with the patient's current situation, specified and staggered, so that the plan can be evaluated in the future.

Clinical conversations and interactions should always end with a question to check if the plan fits with the patient's needs and expectations in order to consolidate and support the change.

## 2 Discussion

A collaborative patient‐healthcare provider therapeutic relationship is a crucial piece in the management of chronic diseases. Since 2019, the patient‐centered European clinical practice guidelines introduced the concept of using motivational interviewing to improve communication and behavior change as well as to avoid stigmatization in a health care setting [77]. These guidelines stated that behavior change is essential for obesity treatment adherence and that this will increase the possibility of patients achieving improved health outcomes based on their values, priorities, and resources. The guideline also recommends that readiness to change (as one of the major determinants of treatment adherence associated with health outcomes) should be evaluated and managed using motivational interviewing (MI). This approach meant a shift in paradigm in the management of obesity, which was later consolidated with the Canadian clinical practice guidelines for obesity management [27].

As discussed earlier, empowering patients to be more involved in their healthcare plans and understanding and accepting patients' perceptions of their own health and illness, may reduce stigma and help healthcare professionals to understand the consequences of stigma on a patients' health and health behaviors, and contribute to a more equitable and collaborative model of obesity care [35]. This has the potential for improving the cost‐effectiveness of obesity care, as has been demonstrated in other chronic disease areas [36].

Studies using MI in obesity management have solely focused on weight loss outcomes. Some studies show efficacy [73, 74] while others do not [75]. However, viewing MI solely through the lens of its effect on weight loss overlooks its primary purpose: facilitating behavior change. Considering that weight and obesity are not behaviors, and that MI isn't designed specifically for managing obesity, it can serve as a powerful tool to empower individuals in altering their health behaviors that are related to their obesity management plans. Therefore, gauging MI’s success solely by weight loss outcomes disregards its fundamental aim and the intricate process of behavior modification. The potential of MI to improve parent/adult caregiver behavior for obesity and cancer prevention rather than BMI changes alone has been studied more recently [78]. This paradigm shift should be accompanied by new research focusing on variables that go beyond weight loss, such as improved self‐efficacy, which involves building confidence in one's ability to make and sustain changes in lifestyle and health‐related behaviors; behavioral changes, such as adopting sustainable habits that contribute to long‐term health, including meal planning, grocery shopping, or cooking at home; psychological outcomes, such as enhancing self‐esteem, reducing stress, improving mood, and decreasing emotional or binge eating patterns; improved health markers, addressing non‐weight‐related outcomes like better blood pressure, cholesterol levels, or blood sugar control; patient engagement, fostering intrinsic motivation for change and empowering individuals to take ownership of their health goals; and quality of life, which encompasses supporting overall well‐being, better sleep, reduced fatigue, and enhanced daily functioning. This broader approach highlights the importance of focusing on diverse health and well‐being outcomes.

On the other hand, the stigma perceived by people living with obesity when interacting with healthcare professionals plays a key role in their experiences of healthcare encounters. Studies demonstrate that healthcare professionals hold explicit (i.e. conscious) and implicit (i.e. unconscious) negative attitudes and beliefs toward patients living with obesity (i.e. weight bias) [12, 25]. A key strategy to reduce weight bias and stigma in healthcare is to identify and change stigmatizing attitudes, practices, and processes among healthcare professionals [27].

The misconception about MI as a strategy to motivate people with obesity to lose weight or to educate people about healthy eating and exercise so that they can lose weight warrants consideration. It is well established that binary “eat less and move more” interventions are not sufficient treatments for obesity [79] and that the simplification of obesity as solely a behavioral problem is stigmatizing [74]. MI is a communication style that helps healthcare professionals and patients understand patients' situation, realities, and barriers for behavior changes that are required to improve health outcomes.

It is worthwhile to consider methods of assessing treatment fidelity in relation to the MI approach when investigating the impact of MI on health outcomes measures in research and clinical practice. It is important to evaluate how treatment fidelity has been assessed, emphasizing the need for repetition and regular supervision in performing MI. Having conversations recorded and coded can be a valuable tool for learning and maintaining MI skills in clinical practice, including the use of specific instruments such as the intra‐class correlation for coders [74, 80]. Examining training and supervision that MI practitioners received prior to and during the study should also be incorporated in the research protocols since these are also important aspects of evidence.

Consistent with the principles and approaches of people‐centered care [81], healthcare providers should adopt a collaborative therapeutic relationship with patients and provide care that is respectful and that can assist patients to make informed treatment and management decisions. It has been proposed that the activities/behaviors (things patient do) for example, participating in shared decision‐making, could be considered immediate outcomes of patient empowerment [82]. Outcomes such as quality of life and social well‐being could be considered intermediate outcomes of patient empowerment, with health status as a possible long‐term outcome. Health literacy is also an indicator of patient empowerment that can be measured because patients need to be able to understand the medical information provided by their healthcare providers to use it effectively in their disease management plans [83].

MI may seem simple, but mastering this technique is neither a quick nor an easy process. It takes training and practice in both the strategies and in often reviewing the relationship we establish with the people we serve. Training health care professionals involved in obesity care will help to change perceptions about obesity and improve overall patient experiences with obesity management. Effective healthcare communications can also support patient empowerment and decrease internalized stigma. Learning Motivational Interviewing is like learning to play an instrument. Initial pointers are important and can help, but learning to play a real instrument takes practice, and if possible, with feedback from expert teachers. As with other complex skills, achieving mastery of MI is a long process, which can last a lifetime. Regular MI training and supervision should be incorporated in obesity education programs as well as in weight stigma education programs for healthcare professionals. Gonat and colleagues have developed a tool to train healthcare professionals on integrating the “focusing” phase of MI into healthcare and public health interventions [84]. This tool could be used in obesity and weight stigma education programs for healthcare professionals.

Adherence to medical therapies is multifactorial. It is now recognized that adherence is not just about patient behavior but that the health systems and healthcare teams can be significant determinants of adherence to medical therapies [85]. Health systems can support adherence to obesity management programs by ensuring that obesity treatments, including behavioral, psychological, and bariatric surgery interventions, are accessible to patients where they live when they need them. Educational institutions can incorporate comprehensive obesity training in healthcare professional curricula, including training on the complexity and chronicity of obesity, evidence‐based treatments, weight bias and stigma, and provide skills training for using MI to nurture collaborative therapeutic relationships with people living with obesity.

Lastly, a fundamental factor of healthcare quality is patient experience, along with safety and effectiveness [86, 87]. Therefore, it is essential that healthcare organizations and professionals identify the unmet needs of patients and families when trying to increase the value and quality of healthcare services [87, 88]. Hancock et al. demonstrated that key factors to improve patient experiences in healthcare include good communication skills (such as active listening) and non‐judgmental attitudes [89]. The principles of collaboration, acceptance, evocation, and compassion, which govern the philosophy of Motivational Interviewing, can support healthcare professionals in having non‐judgmental attitudes and interactions with patients living with obesity.

## 3 Conclusion

Health‐related stigma can lead to inequalities which can affect patients living with chronic conditions such as obesity and prevent help‐seeking and reduce quality of life. Stigma may be both internal and external, often rooted in unconscious bias toward people living with obesity because of their weight. The consequences of stigma (particularly in healthcare settings) are often as harmful as the illness or condition experienced and being aware of this and indeed discussing the consequences of stigma as part of the clinical consultation may provide assurance to patients and facilitate a more open and equitable approach to obesity care.

MI has the potential to explore stigma with patients and may create a structured and supportive environment in which to reframe clinical encounters to provide a more patient‐centered approach to obesity treatments, thereby empowering patients and facilitating positive behavior change, led by the patient. The use of MI in obesity care warrants more research and application in practice.

## Author Contributions

V.M., M.B. prepared the first draft of the manuscript. Y.G. and X.R.S. edited the paper and made major contributions. All authors edited and approved the last version of the manuscript.

## Conflicts of Interest

The authors declare no conflicts of interest.
